# Insulin-like growth factor 2 (IGF-2) rescues social deficits in NLG3^–/*y*^ mouse model of ASDs

**DOI:** 10.3389/fncel.2023.1332179

**Published:** 2024-01-17

**Authors:** Rocco Pizzarelli, Domenico Pimpinella, Christian Jacobs, Alice Tartacca, Uarda Kullolli, Hannah Monyer, Cristina M. Alberini, Marilena Griguoli

**Affiliations:** ^1^European Brain Research Institute (EBRI), Rome, Italy; ^2^Department of Clinical Neurobiology at the Medical Faculty of Heidelberg University and German Cancer Research Center (DKFZ), Heidelberg, Germany; ^3^Center for Neural Science, New York University, New York, NY, United States; ^4^Institute of Molecular Biology and Pathology of the National Council of Research (IBPM-CNR), Rome, Italy

**Keywords:** IGF-2, hippocampus, social behavior, NLG3 -/y mouse, ASDs

## Abstract

Autism spectrum disorders (ASDs) comprise developmental disabilities characterized by impairments of social interaction and repetitive behavior, often associated with cognitive deficits. There is no current treatment that can ameliorate most of the ASDs symptomatology; thus, identifying novel therapies is urgently needed. Here, we used the Neuroligin 3 knockout mouse (NLG3^–/*y*^), a model that recapitulates the social deficits reported in ASDs patients, to test the effects of systemic administration of IGF-2, a polypeptide that crosses the blood-brain barrier and acts as a cognitive enhancer. We show that systemic IGF-2 treatment reverses the typical defects in social interaction and social novelty discrimination reflective of ASDs-like phenotypes. This effect was not accompanied by any change in spontaneous glutamatergic synaptic transmission in CA2 hippocampal region, a mechanism found to be crucial for social novelty discrimination. However, in both NLG3^+/*y*^ and NLG3^–/*y*^ mice IGF-2 increased cell excitability. Although further investigation is needed to clarify the cellular and molecular mechanisms underpinning IGF-2 effect on social behavior, our findings highlight IGF-2 as a potential pharmacological tool for the treatment of social dysfunctions associated with ASDs.

## Introduction

Deficits in social skills are core symptoms of Autism Spectrum Disorders (ASDs). Autistic children exhibit a severe impairment in social abilities: they have hard time in interacting with others and show a reduced attention to social stimuli (McPartland et al., 2011; Pierce et al., 2011). In about 50% of cases these alterations are associated with defects in learning and other cognitive abilities (Hill, 2004; Charman et al., 2011).

Mutations/deletions of genes encoding for synaptic proteins have been found in several forms of ASDs (Zoghbi and Bear, 2012; Guang et al., 2018). Among these, of particular interest are those belonging to neuroligin (NLG)/neurexin (NRX) families. NLGs are postsynaptic adhesion molecules that, by interacting with their presynaptic partners NRXs, functionally couple the postsynaptic densities with the transmitter release machinery, thus contributing to synaptic development and stabilization (Südhof, 2008). Mutations/deletions of X-linked neuroligin 3 (*Nlg3*) gene, have been found in non-syndromic cases of autism (Jamain et al., 2003; Levy et al., 2011; Quartier et al., 2019). The localization of NLG3 at both excitatory and inhibitory synapses is region and circuit specific and depends on its phosporilation state (Varoqueaux et al., 2006; Altas et al., 2022 bioRxiv). Mice carrying the human R451C mutation of the *Nlg3* gene exhibit impaired social approach and enhanced spatial learning associated with alterations of glutamatergic and GABAergic signaling in selective neuronal circuits (Tabuchi et al., 2007; Etherton et al., 2011; Pizzarelli and Cherubini, 2011, 2013; Cellot and Cherubini, 2014). Furthermore, mice lacking NLG3 (NLG3^–/*y*^), show severe alterations in social skills, reminiscent of those observed in autistic children (Radyushkin et al., 2009; Kalbassi et al., 2017; Bariselli et al., 2018; Modi et al., 2019; Hörnberg et al., 2020).

Of the brain areas involved in social behavior, the CA2 region of the hippocampus has recently emerged as a central structure for social memory, namely the capacity of an animal to recognize a conspecific (Hitti and Siegelbaum, 2014; Stevenson and Caldwell, 2014; Meira et al., 2018; Oliva et al., 2020). The CA2 area is characterized by defined molecular, morphological and physiological properties (Mercer et al., 2007; Chevaleyre and Siegelbaum, 2010; Dudek et al., 2016; Chevaleyre and Piskorowski, 2023). In a previous investigation on NLG3^–/*y*^ mice we found that deficits in social memory are associated with a dysfunction of the CA2 network dynamics, consisting of a reduction of gamma power and a severe alteration of both glutamatergic and GABAergic synaptic transmission (Modi et al., 2019). Rescue strategies aiming at restoring physiological activity in neuronal circuits involved in social behavior are needed to cure social deficits affecting autistic children.

Among possible therapeutic candidates, IGF-2, a polypeptide similar to insulin and insulin-like growth factor 1, is a potent cognitive enhancer (Chen et al., 2011), which crosses the blood-brain barrier and produces its effects acting on the IGF-2 high affinity receptors know as IGF-2 receptor (IGF-2R) or cation independent mannose 6 phosphate receptor. IGF-2R is highly enriched in excitatory neurons in all hippocampal subfields (Yu et al., 2020). Recent studies have highlighted the beneficial effects of IGF-2 in ameliorating cognitive deficits in aged-rats, in the BTBR mouse model of ASDs and in the Ube3A maternal-deficient (m-/p +) mouse model of Angelman syndrome (Steinmetz et al., 2016, 2018; Cruz et al., 2021).

Here we show that IGF-2 is effective in ameliorating impaired sociability and social novelty discrimination in NLG3^–/*y*^ mice, as revealed by the three-chamber test. The global effects of IGF-2 on cognitive functions suggest a more generalized type of action that could be possibly extended to other forms of ASDs with different etiologies, making it a promising tool for treating these disorders, for which no therapy is currently available.

## Materials and methods

### Animals

All experiments were performed in accordance with the Italian Animal Welfare legislation (D.L. 26/2014) that were implemented by the European Committee Council Directive (2010/63 EEC) and were approved by local veterinary authorities, the EBRI ethical committee and the Italian Ministry of Health (565/PR18). All efforts were made to minimize animal suffering and to reduce the number of animals used. Mice were housed in 4-5 per cage at constant temperature (22°C) and humidity (30−50%) and were kept on a regular circadian cycle (12 h: 12 h light: dark cycle, lights on at 7:00 a.m.) Mice were provided with food and water *ad libitum*.

We used *NLG3 Knockout* (NLG3^–/*y*^) and wild-type (NLG3^+/*y*^) mice, kindly provided by Prof. Scheiffele (Biozentrum, Basel). Experiments were performed on 6−8 weeks aged male off-spring derived from mating heterozygous females with wild-type males. The experiments were performed and the results were analyzed blindly before genotyping. Genotyping was carried out on tail biopsy DNA by PCR using a standard protocol. At least 4−5 male mice were used for a given experiment.

### Subcutaneous injections in mice

Recombinant mouse IGF-2 (R and D Systems, # 792 MG) was dissolved in 0.1% bovine serum albumin in PBS (BSA-PBS), and 30 mg/kg was injected subcutaneously in the neck region. Vehicle injections consisted of 0.1% BSA-PBS. The dose of 30 μg/kg and the timing of drug delivery were chosen on the basis of previous studies (Stern et al., 2014; Steinmetz et al., 2018; Cruz et al., 2021). Injections were performed 20 min before behavioral test and 1 h and 20 min before slicing procedure.

### Three-chamber test

Sociability and social novelty skills were tested using the three-chamber test, adapted from Moy et al. (2004) in a homemade rectangular, clear Plexiglas three-chambered box (each chamber was 20 × 40 × 21 cm in size). Dividing walls included rectangular openings (6 × 8.5 cm) allowing access to each chamber. The light intensity (6 lux) was distributed equally in the apparatus. Between trials, the chambers of the arena were cleaned with 70% ethanol to eliminate lingering smells. Mice were handled 5 min a day for 5 days before the test. On the day before the test, mice were habituated to the entire empty apparatus for 30 min. On the test day, after a 10 min habituation phase in the entire empty apparatus, the test mouse performed sociability and social novelty trials. During sociability task, the test mouse was placed in the middle compartment and allowed to explore for 10 min between a wire cup (ø 10.5 cm x 10.5 cm h) with an unfamiliar juvenile (P40) C57BL7/6J male mouse (stranger 1) and an identical empty wire cup located in the lateral compartments. The interaction time was recorded by the video-tracking system (ANY-maze, StoeltingCo, IL, US). The measurement of the interaction time started when the head orientation was directed toward the cup (1 cm distance) indicating an active interest of the animal for the object or the conspecifics. After a 1 h inter-trial interval a second unfamiliar C57BL7/6J male mouse (stranger 2 or novel) was placed into the previously empty wire cup, while “stranger 1” (familiar) remained inside its cup for social novelty trial. The test mouse was given 10 min to explore all three chambers. The position of “stranger 1” was alternated between sociability and social novelty trials, to prevent side-preference.

### Slice preparation

Transverse hippocampal slices (320 μm tick) were obtained from postnatal (P) day P45–P60 old animals, using a standard protocol (Bischofberger et al., 2006). Briefly, after being anesthetized with an intraperitoneal injection of a mixture of tiletamine/zolazepam (80 mg/Kg) and xylazine (10 mg/Kg), mice were decapitated. The brain was quickly removed from the skull, placed in artificial cerebrospinal fluid (ACSF) containing (in mM): sucrose 75, NaCl 87, KCl 2.5, NaH_2_PO_4_ 1.25, MgCl_2_ 7, CaCl_2_ 0.5, NaHCO_3_ 25, glucose 25. After recovery, an individual slice was transferred to a submerged recording chamber and continuously perfused at room temperature with oxygenated ACSF at a rate of 3 ml/min. ASCF saturated with 95% O_2_ and 5% CO_2_ and contained in mM: NaCl 125, KCl 2.5, NaH_2_PO_4_ 1.25, MgCl_2_ 1, CaCl_2_ 2, NaHCO_3_ 25, glucose 10.

### Patch clamp recordings in slices

Cells were visualized with a 60X water immersed objective mounted on an upright microscope (Nikon, eclipse FN1) equipped with a CCD camera (Scientifica, UK). Whole-cell patch clamp recordings, in voltage and current clamp modes, were performed with a MultiClamp 700B amplifier (Axon Instruments, Sunnyvale, CA, USA). Patch electrodes were pulled from borosilicate glass capillaries (Hilgenberg, Germany); they had a resistance of 3−4 MΩ when filled with an intracellular solution containing (in mM): K gluconate 70, KCl 70, HEPES 10, EGTA 4, MgCl_2_ 2, MgATP 4, MgGTP 0.3, Na-phosphocreatine 5; the pH was adjusted to 7.2 with KOH; the osmolarity was 295−300 mOsm. Membrane potential values were not corrected for a liquid junction potential.

Spontaneous AMPA-mediated excitatory postsynaptic currents (sEPSCs) were recorded from pyramidal neurons in the CA2 region of the hippocampus whose membrane potential was held at −70 mV in the presence of picrotoxin (100 μM). Pyramidal neurons were identified in current-clamp mode by their passive and active mebrane properties as reported previously (Chevaleyre and Siegelbaum, 2010). Spiking activity was evoked by applying depolarizing current steps (50 pA increments, 800 ms duration) from a holding potential of −65 mV. The sag in the electrotonic potentials, triggered by hyperpolarization-activated current I_*h*_, was obtained by injecting steady hyperpolarizing currents into the cell (−150 pA, 800 ms) from a holding potential of −65 mV. The stability of the patch was checked by repetitively monitoring the input and series resistance during the experiments. Series resistance (10−20 MΩ) was not compensated. Cells exhibiting 20% changes were excluded from the analysis. Picrotoxin was applied in the bath and the ratio of flow rate to bath volume ensured complete exchange within 3−4 min.

### Data analysis and statistics

Electrophysiological data were transferred to a computer hard disk after digitization with an A/D converter (Digidata 1550, Molecular Devices, Sunnyvale, CA, USA). Data acquisition (digitized at 10 kHz and filtered at 3 kHz) was performed with pClamp 10.4 software (Molecular Devices, Sunnyvale, CA, USA). Input resistance and cells capacitance were measured online with the membrane test feature of the pClamp software. Spontaneous EPSCs were analyzed with pClamp 10.4 (Molecular Devices, Sunnyvale, CA, USA). This program uses a detection algorithm based on a sliding template. The template did not induce any bias in the sampling of events because it was moved along the data trace by one point at a time and was optimally scaled to fit the data at each position. Spikes evoked by incremental current steps were counted manually. The sag was measured from the normalized steady state to peak voltage response of the cell to a hyperpolarizing current step and defined as follows: 100 x (1 - V_*ss*_/V_*peak*_), where V_*ss*_ is the steady-state voltage deflection from baseline, and V_*peak*_ is the peak voltage deflection from baseline (Narayanan and Johnston, 2007). The input resistance (R_*in*_) was calculated as described previously (Mao et al., 2006). Briefly, the V_*ss*_ and V_*peak*_ values were obtained from hyperpolarizing steps (−50 pA increment, 800 ms). Rinput was calculated from the average slope of the voltage-current (V-I) curve extrapolated for both V_*peak*_-I and Vss-I curves.

Behavioral data analysis was performed manually and the experimenter was blinded to the genotype but not to the treatment. A second experimenter repeated the analysis in blind and the results obtained were comparable. The sociability discrimination index was calculated as the ratio between the difference among the investigation time for the object and that for the animal, and the total time of interaction. The social novelty discrimination index was calculated as the ratio between the difference among the investigation time for the familiar and that for the novel mouse, and the total time of interaction (Rein et al., 2020).

Details of specific statistical designs and appropriate tests are described in the result section and in each figure legend. Values are given as the mean ± SEM of n experiments. No statistical methods were used to predetermine sample sizes, but our samples were in agreement with similar published studies. All datasets were tested for normality using Shapiro–Wilk test. Data that passed normality test were compared using two-way ANOVA followed by Tukey’s test for multiple comparisons. Data that failed normality test were compared using Kruskall-Wallis, followed by *post-hoc* Dunn’s test for multiple comparisons within and between groups. Outliers were identified using ROUT method (*Q* = 1%). Statistical differences were considered significant at *p* < 0.05. Statistical analysis was performed with GraphPad Prism 8.0 software (GraphPad, CA, US).

### Drugs

Picrotoxin, purchased from Tocris (UK), was applied in the bath by gravity by changing the superfusion solution to one differing only in its content of drug. Stock solution of picrotoxin was made in DMSO. Aliquots were then frozen at −20°C. The final concentration of DMSO in the bathing solution was 0.1%. At this concentration, DMSO alone did not modify the membrane potential, input resistance or the firing properties of CA2 neurons.

## Results

### IGF-2 reverses social deficits of NLG3^–/*y*^ mice

**FIGURE 1:**
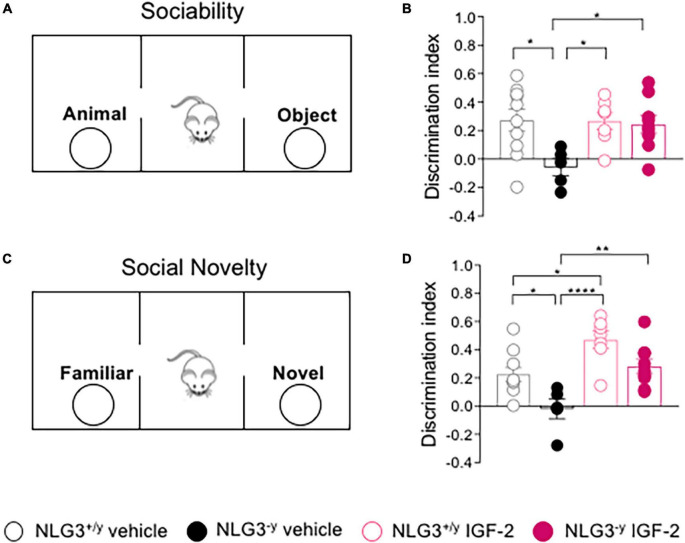
IGF-2 reverses social deficits of NLG3-/y mice. **(A)** Schematic representation of the three-chamber paradigm (modified by Pimpinella et al., 2021) used to evaluate sociability. **(B)** Aligned dot plots showing sociability discrimination index in NLG3^ + ⁣/y^ and NLG3^−⁣/y^ mice treated with vehicle or IGF-2. **(C)** Schematic representation of the three chamber paradigm used to evaluate social novelty. **(D)** Aligned dot plots showing social novelty discrimination index evaluated 1 hour after sociability in NLG3^ + ⁣/y^ and NLG3^−⁣/y^ mice treated with vehicle or IGF-2. Bars represent the average SEM. Open and filled circles represent values from single animals. *p < 0.05, **p < 0.01, ****p < 0.0001, two-way ANOVA followed by multiple comparison Tukey’s test.

Previous studies from our laboratory and others demonstrated that NLG3^–/*y*^ mice exhibit deficits in both social interaction and social novelty (Radyushkin et al., 2009; Bariselli et al., 2018; Modi et al., 2019; Hörnberg et al., 2020). Here, we tested the effect of subcutaneous administration of IGF-2 to adult male NLG3^–/*y*^ mice and compared them to NLG3^+/*y*^ littermates. For all experiments, the dose of 30 μg/kg and the timing of drug delivery were chosen on the basis of previous studies (Stern et al., 2014; Steinmetz et al., 2018; Cruz et al., 2021). 20 min after the injection, the animals were subjected to the three chamber test for both sociability and social novelty evaluation (Figures 1A, C). According to our previous work on non-treated animals (Modi et al., 2019), NLG3^–/*y*^ injected with vehicle showed impaired sociability as compared to NLG3^+/*y*^ whereas NLG3^–/*y*^ mice treated with IGF-2 behaved as NLG3^+/*y*^ mice showing a preference for the animal over the object (Figure 1B; sociability discrimination index of NLG3^+/*y*^ *vs.* NLG3^–/*y*^ treated with vehicle: 0.27 ± 0.08 *vs.* −0.06 ± 0.06, *n* = 10 *vs. n* = 5; sociability discrimination index of NLG3^+/*y*^ *vs.* NLG3^–/*y*^ treated with IGF-2: 0.27 ± 0.06 *vs.* 0.24 ± 0.06; *n* = 7 *vs. n* = 9; F_1_,_27__(*treatment*) =_ 4.14, *p* = 0.04, F_1_,_27__(*genotype*) =_ 5.86, *p* = 0.05 F_1_,_27__(*treatment x genotype*)_ = 4.48, *p* = 0.04, two-way ANOVA followed by Tukey’s test for comparisons). To evaluate social novelty discrimination, after 1 h the test mouse was subjected to a social novelty task consisting in the exposition to a previously encountered familiar mouse and a novel one. IGF-2-treated NLG3^–/*y*^ mice showed the typical preference for the novel subject compared to the familiar one, as was the case in NLG3^+/*y*^ group indicating that IGF-2 administration was effective in reverting the deficits in social novelty discrimination observed in NLG3^–/*y*^ treated with vehicle (Figure 1D; social novelty discrimination index of NLG3^+/*y*^ *vs.* NLG3^–/*y*^ treated with vehicle: 0.22 ± 0.05 *vs.* −0.19 ± 0.07, *n* = 10 *vs. n* = 5; social novelty discrimination index of NLG3^+/*y*^ *vs.* NLG3^–/*y*^ treated with IGF-2: 0.49 ± 0.06 *vs.* 0.28 ± 0.05; *n* = 7 *vs. n* = 9; F_1_,_27__(*treatment*) =_ 22.0, *p* < 0.0001, F_1_,_27__(*genotype*)_ = 13.7,*p*=0.001 F_1_,_27__(*treatment x genotype*)_ = 0.22, *p* = 0.64, two-way ANOVA followed by Tukey’s test for comparisons). Moreover IGF-2 significantly increased social novelty skills of NLG3^+/*y*^ as compared to vehicle-treated NLG3^+/*y*^ confirming its role as a cognitive enhancer (Chen et al., 2011).

### IGF-2 does not affect glutamatergic synaptic transmission in the CA2 hippocampal region

**FIGURE 2:**
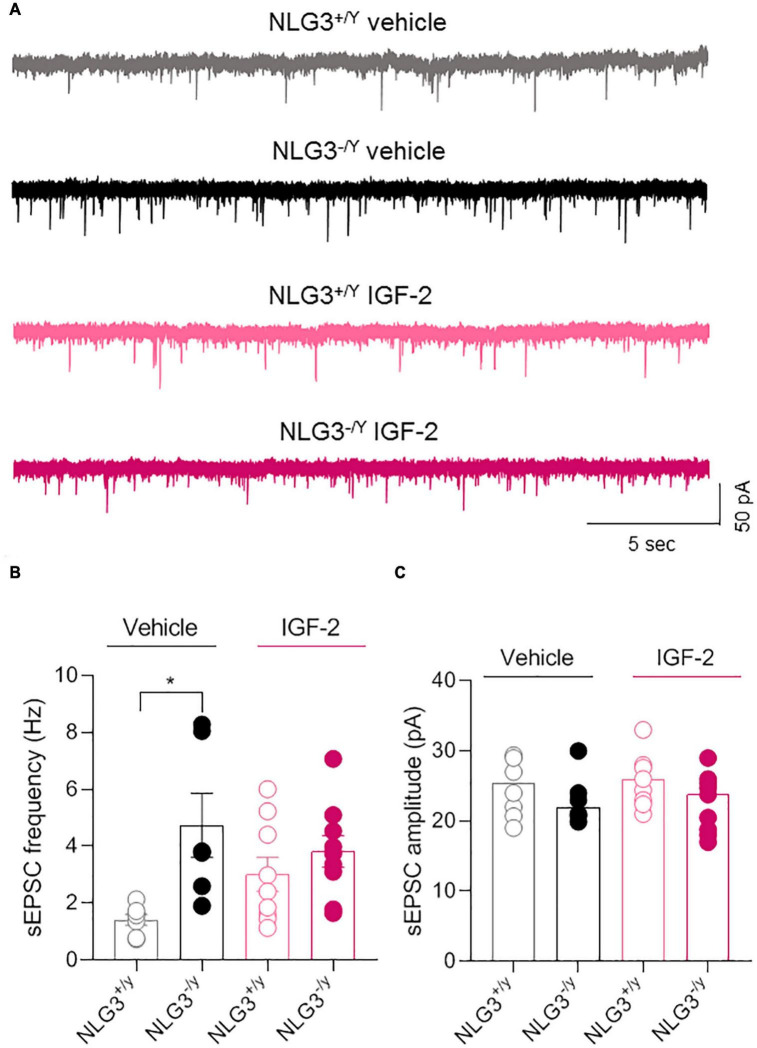
IGF-2 does not affect glutamatergic synaptic transmission in the CA2 hippocampal region. **(A)** Sample traces showing sEPSCs recorded from CA2 pyramidal neurons in hippocampal slices obtained from NLG3^+/*y*^ and NLG3^–/*y*^ mice treated with vehicle or IGF-2. **(B)** Aligned dot plot showing the mean sEPSCs frequency of sEPSCs recorded from CA2 pyramidal neurons of NLG3^+/*y*^ and NLG3^–/*y*^ mice treated with vehicle or IGF-2. **(C)** Aligned dot plot showing the mean sEPSCs amplitude of sEPSCs recorded from CA2 pyramidal neurons of NLG3^+/*y*^ and NLG3^–/*y*^ mice treated with vehicle or IGF-2. Bars represent the average ± SEM. Open and filled circles represent values from single cells. *p < 0.05, two-way ANOVA followed by multiple comparison Tukey’s test.

The CA2 region of the hippocampus is part of the so called “social brain”, i.e., complex network of brain regions responsible for social cognition. In particular, the CA2 area has been demonstrated to play a key role in social memory formation (Hitti and Siegelbaum, 2014; Stevenson and Caldwell, 2014; Meira et al., 2018; Oliva et al., 2020). Interestingly, altered CA2 neuronal activity was found in animal models of neurodevelopmental disorders like ASDs and schizophrenia (Piskorowski et al., 2016; Modi et al., 2019; Donegan et al., 2020) or in sclerotic hippocampal tissue surgically excised from patients affected by temporal lobe epilepsy (Wittner et al., 2009). To determine whether IGF-2-mediated rescue of social novelty was accompanied by a recovery of the synaptic transmission in the CA2 region, we used the patch clamp technique to record spontaneous postsynaptic excitatory currents (sEPSCs), in the presence of picrotoxin in the extracellular solution to block GABAergic transmission from CA2 pyramidal neurons of NLG3^+/*y*^ and NLG3^–/*y*^ mice treated with vehicle or IGF-2, using the same paradigm of administration employed for behavioral experiments. In agreement with a previous study (Modi et al., 2019), we observed an enhanced sEPSC frequency in NLG3^–/*y*^ mice as compared to NLG3^+/*y*^ animals treated with vehicle. However, following IGF-2 treatment no significant changes in sEPSCs frequency were detected (Figures 2A, B, sEPSCs frequency of NLG3^+/*y*^ *vs.* NLG3^–/*y*^ treated with vehicle: 1.4 ± 0.2 Hz *vs.* 4.7 ± 1.1 Hz, *n* = 7 *vs. n* = 6; sEPSCs frequency of NLG3^+/*y*^ *vs.* NLG3^–/*y*^ treated with IGF-2: 3.0 ± 0.6 Hz *vs.* 3.8 ± 0.5 Hz, *n* = 9 *vs. n* = 9; F_1_,_27__(*treatment*)_ = 0.26, *p* = 0.61, F_1_,_27__(*genotype*)_ = 9.19, *p* = 0.004, F_1_,_27__(*treatment*_ _*x genotype*)_ = 3.68, *p* = 0.07, two-way ANOVA followed by Tukey’s test for comparisons). No changes in sEPSCs amplitude were observed among all experimental groups (Figures 2A, C), sEPSCs amplitude of NLG3^+/*y*^ *vs.* NLG3^–/*y*^ treated with vehicle: 25 ± 1.5pA *vs.* 23.1 ± 1.5pA, *n* = 7 *vs. n* = 6; sEPSCs amplitude of NLG3^+/*y*^ vs. NLG3^–/*y*^ treated with IGF-2: 25.7 ± 1.2pA *vs.* 22.6 ± 1.4pA, *n* = 9 *vs. n* = 9; F_1_,_27__(*treatment*)_ = 0.08, *p* = 0.78, F_1_,_27__(*genotype*)_ = 2.43, *p* = 0.13, F_1_,_27__(*treatment x genotype*)_ = 0.42, *p* = 0.52, two-way ANOVA followed by Tukey’s test for comparisons). These results suggest that IGF-2 does not affect spontaneous glutamatergic synaptic transmission.

### IGF-2 enhances CA2 neuronal excitability

**FIGURE 3:**
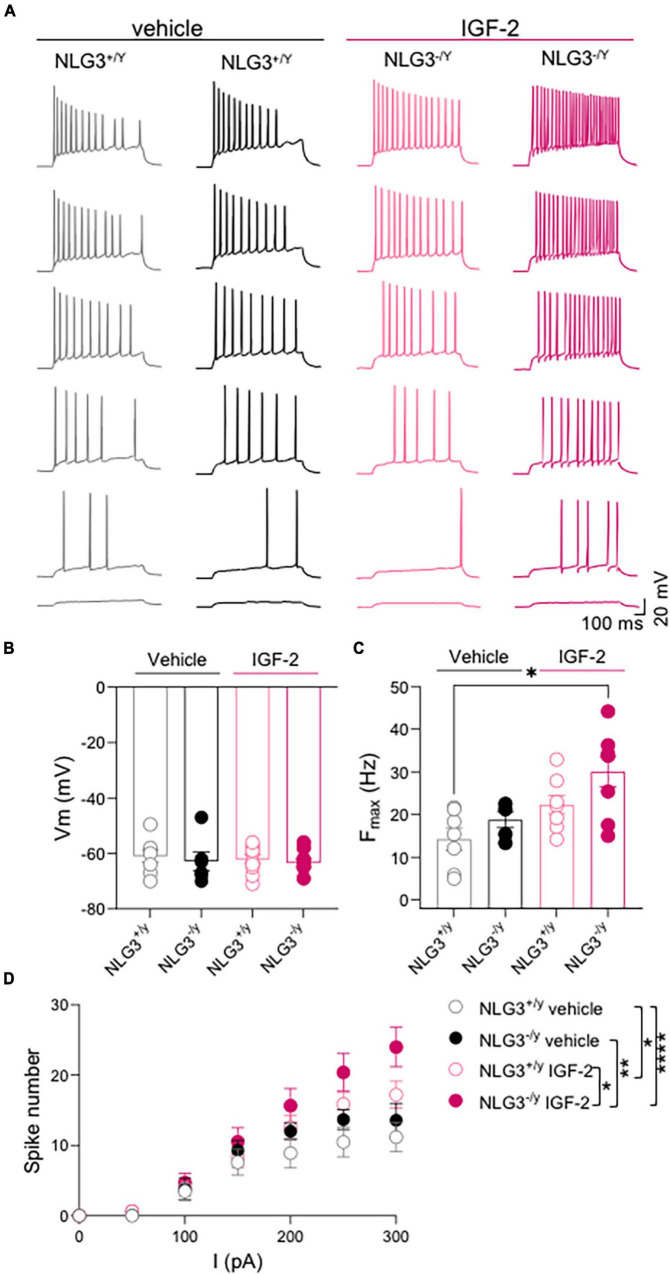
IGF-2 enhances CA2 neuronal excitability. **(A)** Sample traces of voltage responses evoked by depolarizing current steps recorded from CA2 pyramidal neurons in hippocampal slices obtained from NLG3^+/*y*^ and NLG3^–/*y*^ mice treated with vehicle or IGF-2. **(B)** Aligned dot plots showing the resting membrane potential of CA2 pyramidal neurons from NLG3^+/*y*^ and NLG3^–/*y*^ mice treated with vehicle or IGF-2. **(C)** Aligned dot plots showing the maximal spiking frequency evoked by depolarizing current steps applied to CA2 pyramidal neurons of NLG3^+/*y*^ and NLG3^–/*y*^ mice treated with vehicle or IGF-2. **(B–C)** *p < 0.05, Kruskal-Wallis test followed by multiple comparison Dunn’s test; **(D)** Dot plot showing the spikes number obtained in response to depolarizing current steps recorded from CA2 pyramidal neurons of NLG3^+/*y*^ and NLG3^–/*y*^ mice treated with vehicle or IGF-2. Bars represent the average ± SEM. Open and filled circles represent values from single cells. *p < 0.05, **p < *0.01*, ****p < 0.0001 two-way ANOVA followed by multiple comparison Tukey’s test.

To understand whether IGF-2 may have an effect on intrisinc neuronal properties we analyzed passive and active membrane parameters in the experimental groups. No difference in resting membrane potential, measured soon after breaking the membrane in the patch clamp, were observed among the groups (Figure 3B; V_*m*_ of NLG3^+/*y*^ *vs.* NLG3^–/*y*^ treated with vehicle: −60.9 ± 2.2 mV *vs.* −62.8 ± 3.4 mV, *n* = 8 *vs. n* = 6; V_*m*_ of NLG3^+/*y*^ vs. NLG3^–/*y*^ treated with IGF-2: −62.1 ± 1.6 mV *vs.* −63.4 ± 1.5 mV, *n* = 10 *vs. n* = 9; *p* = 0.77 Kruskal-Wallis test followed by Dunn’s test for comparisons).

Depolarizing current steps were delivered to CA2 pyramidal neurons to characterize their firing properties including the maximal firing frequency and the spike number (Figures 3A, C, D) in the presence of picrotoxin in the extracellular solution to block GABAergic transmission. While no differences were detected between vehicle treated-NLG3^+/*y*^ and -NLG3^–/*y*^, IGF-2 treatment significantly increased the maximal firing frequency of NLG3^–/*y*^ neurons when compared to those from vehicle-treated NLG3^+/*y*^ mice (Figure 3C; F_*max*_ of NLG3^+/*y*^ *vs.* NLG3^–/*y*^ treated with vehicle: 14.2 ± 2.6 Hz *vs.* 18.7 ± 1.8 Hz, *n* = 7 *vs. n* = 5; F_*max*_ of NLG3^+/*y*^ vs. NLG3^–/*y*^ treated with IGF-2: 22.3 ± 2.1 Hz *vs.* 30 ± 3.5 Hz, *n* = 8 *vs. n* = 8; *p* = 0.02, Kruskal-Wallis test followed by Dunn’s test for comparisons).

**FIGURE 4:**
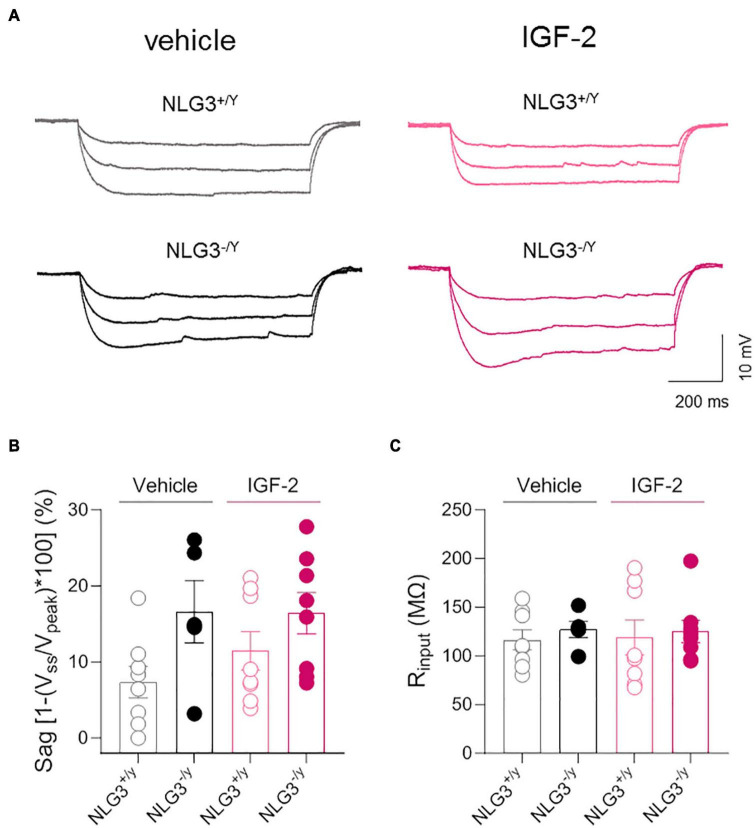
IGF-2 does not affect membrane potential sag and input resistance of CA2 pyramidal neurons. **(A)** Sample traces of voltage responses evoked by hyperpolarizing current steps recorded from CA2 pyramidal neurons of NLG3^+/*y*^ and NLG3^–/*y*^ mice treated with vehicle or IGF-2. **(B)** Aligned dot plot showing the voltage sag [1-(V_*ss*_/V_*peak*_)*100] evoked by hyperpolarizing current step in CA2 pyramidal neurons of NLG3^+/*y*^ and NLG3^–/*y*^ mice treated with vehicle or IGF-2. **(C)** Aligned dot plot showing the input resistance of CA2 pyramidal neurons of NLG3^+/*y*^ and NLG3^–/*y*^ mice treated with vehicle or IGF-2. Bars represent the average ± SEM. Open and filled circles represent values from single cells. Kruskal-Wallis test followed by multiple comparison Dunn’s test.

To further investigate the effect of IGF-2 on spiking behavior, an input/output curve was obtained by counting the spike number evoked by depolarizing current steps. This analysis revealed no difference among spike number evoked by increasing current injections in NLG3^+/*y*^ and NLG3^–/*y*^ animals treated with vehicle but revealed a potentiating effect of IGF-2 treatment in both genotypes (Figure 3D, total spike number of NLG3^+/*y*^ *vs.* NLG3^–/*y*^ treated with vehicle: 5.9 ± 1.8 *vs.* 7.5 ± 2.3, *n* = 7 *vs. n* = 5; total spike number of NLG3^+/*y*^ *vs.* NLG3^–/*y*^ treated with IGF-2: 8.4 ± 2.7 *vs.* 10.8 ± 3.6, *n* = 8 *vs. n* = 8; F (18, 168) = 1.797; F_18_,_168__(*current injected*)_ = 64.8, *p* = < 0.0001, F_18_,_168__(*genotype–treatment*)_ = 11.2, *p* = < 0.0001, F_18_,_168__(*current injected* x genotype–treatment)_ = 1.79, *p* = 0.03, two-way ANOVA followed by Tukey’s test for comparisons). These data are in line with another study focused on CA1 pyramidal neurons showing that IGF-2 binding protein increases spiking activity (Khan et al., 2019). To understand whether the IGF-2-induced change in neuron excitability was due to changes in the pacemaker current Ih, hyperpolarizing current steps were delivered to activate the time-dependent inward rectifying cationic current Ih revealed by a sag in the membrane voltage deflection. No significant differences were detected between NLG3^–/*y*^ and NLG3^+/*y*^ mice treated with either vehicle or IGF-2 (Figures 4A, B, sag of NLG3^+/y^ *vs.* NLG3^–/y^ treated with vehicle: 7.3 ± 2.1% *vs.* 16.6 ± 4.1%, *n* = 8 *vs. n* = 5; sag of NLG3^+/y^ *vs.* NLG3^–/y^ treated with IGF-2: 3.9 ± 2.5% *vs.* 16.4 ± 2.7%, *n* = 8 *vs. n* = 8; *p* = 0.11, Kruskal-Wallis test followed by Dunn’s test for multiple comparisons) indicating that Ih current is not responsible for IGF-2-induced increase in the spike number. To study whether IGF-2-induced changes in spike number were due to differences in background membrane conductances we also measured the input resistance from the voltage deflection evoked by hyperpolarizing current steps (see method section). No differences were detected among all experimental groups (Figure 4C, R_*input*_ of NLG3^+/y^ *vs.* NLG3^–/y^ treated with vehicle: 116 ± 10 MΩ *vs.* 127 ± 8 MΩ, *n* = 8 *vs. n* = 5; R_*input*_ of NLG3^+/y^ *vs.* NLG3^–/y^ treated with IGF-2: 119 ± 18 MΩ *vs.* 125 ± 11 MΩ, *n* = 8 *vs. n* = 8; *p* = 0.81, Kruskal-Wallis test followed by Dunn’s test for comparisons).

## Discussion

As previous studies provide evidence that IGF-2 is effective in rescuing social deficits occurring in mice models of neurodevelopmental disorders (Steinmetz et al., 2018; Cruz et al., 2021), we sought to test the effectiveness of IGF-2 in treating social deficits of the NLG3^–/y^ mice, a monogenic model of ASDs (Baudouin et al., 2012). The data revealed that NLG3^–/y^ mice treated with IGF-2 fully recovered both the sociability and social novelty skills, that were tested with the three-chamber behavioral paradigm. IGF-2 binds to IGF-2R, which are differentially expressed by neurons in different brain regions including the hippocampus (Hawkes and Kar, 2003; Fushimi et al., 2004; Yu et al., 2020).

In a previous study, we showed that social deficits in NLG3^–/y^ mice were associated with an enhanced frequency of spontaneous glutamatergic postsynaptic currents (sEPSCs) recorded from pyramidal neurons of the CA2 region of the hippocampus, a brain area necessary for social novelty discrimination (Hitti and Siegelbaum, 2014; Stevenson and Caldwell, 2014; Meira et al., 2018; Oliva et al., 2020). To understand whether the effectiveness of IGF-2 in treating social deficits of NLG3^–/y^ mice was associated with normalization of glutamatergic transmission to NLG3^+/y^ levels, we recorded sEPSCs in CA2 pyramidal neurons from NLG3^–/y^ and NLG3^+/y^ mice previously treated with IGF-2 or its vehicle. IGF-2 treatment did not significantly change the frequency or the amplitude of sEPSCs in both NLG3^–/y^ and NLG3^+/y^ mice suggesting that the drug did not target basal glutamatergic transmission in CA2 hippocampal area. This finding is in accordance with a previous study showing no changes in basal synaptic transmission in CA1 pyramidal neurons in IGF-2-perfused slices, obtained from wild type animals (Chen et al., 2011).

In contrast, virus-mediated overexpression of IGF-2 rescues the frequency of miniature EPSCs in the CA1 region in a mouse model of Alzheimer Disease as compared to control mice (Pascual-Lucas et al., 2014). The difference among these studies may be due to different circulating IGF-2 levels that may be reached when using different approaches or animal models (ASDs vs. AD), and that ultimately may involve different neuronal circuits.

Our results do not exclude the possibility that IGF-2 may act on synaptic plasticity and future experiments are needed to clarify this point. In this regard, previous studies point to a permissive role of IGF-2 for synaptic plasticity induction in both mouse and *Aplysia* (Chen et al., 2011; Kukushkin et al., 2019). Molecular mechanisms of IGF-2 action are still poorly understood. Previous studies on the memory-enhancing effects of IGF-2 in both rats injected intra-hippocampus and mice injected *s.c.* showed that the effect requires IGF-2R and not IGF-1R in the hippocampus (Chen et al., 2011; Steinmetz et al., 2018). Similar data were found in the ASD model BTBR, where the positive effects of *s.c.* injection of IGF-2 in reversing impairments in memory and social interaction required hippocampal IGF-2R and not IGF-1R (Steinmetz et al., 2018), leading us to hypothesize that the IGF-2 effect in NLG3^–/y^ mice may also take place *via* IGF-2R. The effects of IGF-2 in rats and mice on either memory enhancement or the recovery of behavioral impairments in mouse models of neurodevelopmental disorders (ASD and Angelman syndrome) is very rapid and, in fact, are detected by 20 min after injection. This rapid effect is consistent with an IGF-2R action because this receptor contributes to endosomal trafficking and regulates lysosomal targeting and cellular functions that occur very quickly (Ghosh et al., 2003). In agreement, blocking IGF-2R in the hippocampus affects memory formation within minutes (Yu et al., 2020). On the basis of the current knowledge, an hypothetical model for the action of IGF-2 and IGF-2R in memory and neurobiological diseases has been proposed (Alberini, 2023). IGF-2R expression has been investigated with immunohistochemistry in the hippocampus of both rats and mice and found to be mostly enriched in neurons, where it is required for *de novo* protein synthesis induced by learning, a fundamental requirement for long-term memory formation (Yu et al., 2020; Alberini, 2023). Despite the current understanding suggests the hypothesis that the effect of IGF-2 on NLG3^–/y^ mice may take place via IGF-2R, the precise mechanisms of action remain to be investigated. As the effect of IGF-2 is contingent upon behavioral activation (Chen et al., 2011) and our electrophysiological experiments were performed in non-trained animals, the lack of rescuing effect of IGF-2 on spontaneous glutamatergic transmission in CA2 could be explained by the lack of an activity-dependent state.

Furthermore, as IGF-2 binding protein, regulating IGF-2 levels, seems to modulate GABAergic transmission and plasticity (Khan et al., 2019) we cannot exclude that in our experiments IGF-2 may modulate GABAergic signalling in CA2.

We next evaluated the effect of IGF-2 on spiking activity of CA2 pyramidal neurons. IGF-2 increases the spike number and the maximal firing frequency in response to depolarizing current steps in both NLG3^–/y^ and NLG3^+/y^ mice. The enhanced excitability is not associated with differences in sag potentials, deflections of the membrane voltage evoked by hyperpolarizing current steps, suggesting that Ih pacemaker current is not regulated by IGF-2.

Further investigation is needed to identify the IGF-2-triggered mechanisms responsible for social deficits rescue in NLG3^–/y^ and those controlling neuronal excitability targeted by IGF-2 in both NLG3^–/y^ and NLG3^+/y^ mice. Future electrophysiological experiments aiming to study the effect of IGF-2 on both glutamatergic and GABAergic synaptic transmission, and plasticity will be performed on trained animals. Biochemical experiments will be also employed to elucidate the molecular mechanisms behind the IGF-2-induced rescue of social deficits in NLG3^–/y^ mice.

## Data availability statement

The raw data supporting the conclusions of this article will be made available by the authors, without undue reservation.

## Ethics statement

The animal study was approved by the EBRI ethical committee and the Italian Ministry of Health (565/PR18). The study was conducted in accordance with the local legislation and institutional requirements.

## Author contributions

RP: Data curation, Formal analysis, Investigation, Supervision, Writing – review & editing. DP: Data curation, Formal analysis, Investigation, Writing – review & editing. CJ: Formal analysis, Investigation, Writing – review & editing. AT: Formal analysis, Investigation, Writing – review & editing. UK: Writing – review & editing. HM: Funding acquisition, Writing – review & editing. CA: Resources, Writing – review & editing. MG: Conceptualization, Formal analysis, Funding acquisition, Investigation, Methodology, Supervision, Writing – original draft, Writing – review & editing.
